# Association between Geriatric Nutritional Risk Index and all-cause mortality in individuals with osteoporotic fractures: a retrospective cohort study

**DOI:** 10.1007/s40520-025-02978-w

**Published:** 2025-03-11

**Authors:** Ming-xin Chen, Li-long Feng, Ke Lu, Chong Li, Yin-lin Wei, Jian Jin, Wen-bin Hu, Yue-qin Guo, Hui-qiang Shan

**Affiliations:** 1 Department of Orthopedics, Affiliated Kunshan Hospital of Jiangsu University, No. 566 East of Qianjin Road, Suzhou, 215300 Jiangsu China; 2 Kunshan Municipal Health and Family Planning Information Center, Suzhou, Jiangsu China; 3 Chronic Disease Department, Kunshan Center for Disease Control and Prevention, Suzhou, Jiangsu China; 4 Department of Endocrinology, Kunshan Fifth People’s Hospital, Suzhou, Jiangsu China

**Keywords:** Geriatric Nutritional Risk Index, GNRI, Mortality, Osteoporotic fractures

## Abstract

**Background:**

The number of patients with osteoporotic fractures (OPFs) is on the rise because of global aging. However, few studies have examined the connection between Geriatric Nutritional Risk Index (GNRI) and overall mortality among inpatients with OPFs. Thus, our research seeks to investigate the link between GNRI and overall mortality in inpatients with OPFs.

**Methods:**

A retrospective cohort study was investigated on 3143 Kunshan OPFs residents aged ≥ 50 years. Participants were stratified into malnutrition (GNRI ≤ 98) and no malnutrition groups (GNRI > 98). Multivariate Cox regression analyses were utilized to evaluate the connection between GNRI and overall mortality. No non-linear association was detected through smoothed curve fitting and threshold analysis. Kaplan–Meier curves were employed to compare the cumulative risk of mortality across varying nutritional conditions. Subgroup analyses were conducted to further investigate the effects of age, sex and other clinical and laboratory factors on the link between GNRI and mortality.

**Results:**

3,143 qualified inpatients with OPFs were involved in the final evaluation. Kaplan–Meier curves revealed that the cumulative risk of mortality was markedly elevated in malnutrition group compared to no malnutrition group. In complete adjustments model, malnutrition group showed an adjusted hazard ratio (aHR) of 1.42 [95% CI 1.05, 1.90; *P*-value = 0.021]. Furthermore, subgroup analyses revealed that no substantial interactions were detected among all variables. (*P-*interaction > 0.05).

**Conclusions:**

Reduced GNRI scores are linked to higher mortality in inpatients with OPFs. The GNRI potentially serve as a predictor for overall mortality risk in this population.

**Supplementary Information:**

The online version contains supplementary material available at 10.1007/s40520-025-02978-w.

## Introduction

Osteoporosis is the most widespread metabolic bone condition [1]. In 2015, individuals aged 50 and older in China were 34.65% more likely to suffer from osteoporosis [2]. This disorder is characterized by diminished bone density and changes in bone microstructure, which elevate the risk of fractures [3]. Fractures represent the most severe consequence of osteoporosis, resulting in significant healthcare expenses, disability, and death [4].

OPFs also known as fragility fractures (FFs), are breaks that happen due to low-energy falls from a standing position or less [5]. As the population ages, the frequency of these fractures is progressively rising around the globe [6]. In an elderly population, it is projected that an osteoporotic fracture (OPF) happens every three seconds, with around 50% of women and 20% of men suffering their first osteoporotic fracture after age 50 [7]. The impact of OPFs is profound, as they are associated with increased death rates, rising medical expenses, and severe consequences such as reduced mobility, persistent pain, disability, loss of self-sufficiency, and diminished quality of life [8]. As a result, OPFs have emerged as a major public health issue. Furthermore, the present study shows that individuals aged 50 and older who have suffered an OPF are at a high risk of experiencing additional fractures [9].

Recent research has shown that malnutrition can play a role in the onset of osteoporosis, thereby heightening the likelihood of fractures [10]. Unlike other clinical variables, malnutrition is an adjustable risk factor that can be addressed by healthcare providers. Malnutrition poses a major issue in hospital environments, affecting 30% to 50% of admitted patients, either already experiencing malnutrition or at risk of its development [11]. The GNRI serves as a measure of nutritional health. As far as we know, a comprehensive examination of the relationship between the GNRI levels and overall mortality in hospitalized patients with OPFs has not been conducted. Therefore, this research employed inpatient records from our hospital from 2018 and 2023 to explore the connection between the GNRI levels and overall mortality among hospitalized patients with OPFs.

## Materials and methods

### Data origin

We retrieved the digital medical records of patients aged 50 and older who lived in Kunshan and had recently received a diagnosis of OPF, requiring hospital admission for surgical treatment. In addition, these admitted patients had been free of any fractures for a minimum of five years, which led to their classification as first-time OPF cases. The fractures analyzed in the study were located at the wrist, upper part of the humerus, hip, and vertebrae. Common sites for OPFs, often termed major OPFs, include the spine, hip, distal radius, and proximal humerus. These fractures were identified according to the tenth edition of the International Statistical Classification of Diseases and Related Health Problems (ICD-10), using codes that start with S22, S32, S42, S52, or S72. In this research, we performed a retrospective cohort study on patient records gathered between November 29, 2018, and August 21, 2023. The research enrolled patients with initial OPFs who were sequentially admitted to Kunshan Hospital, which is associated with Jiangsu University. This study acquired ethical clearance from the AKHJU (approval number: 2024-03-053-H00-K01) and adhered to the guidelines of the Helsinki Declaration. The researchers analyzing the data were blinded to the patient-identifiable information. This is an observational study and informed permission as well as anonymized data was acquired from all the patients.

### Study design and participants

**Fig. 1:**
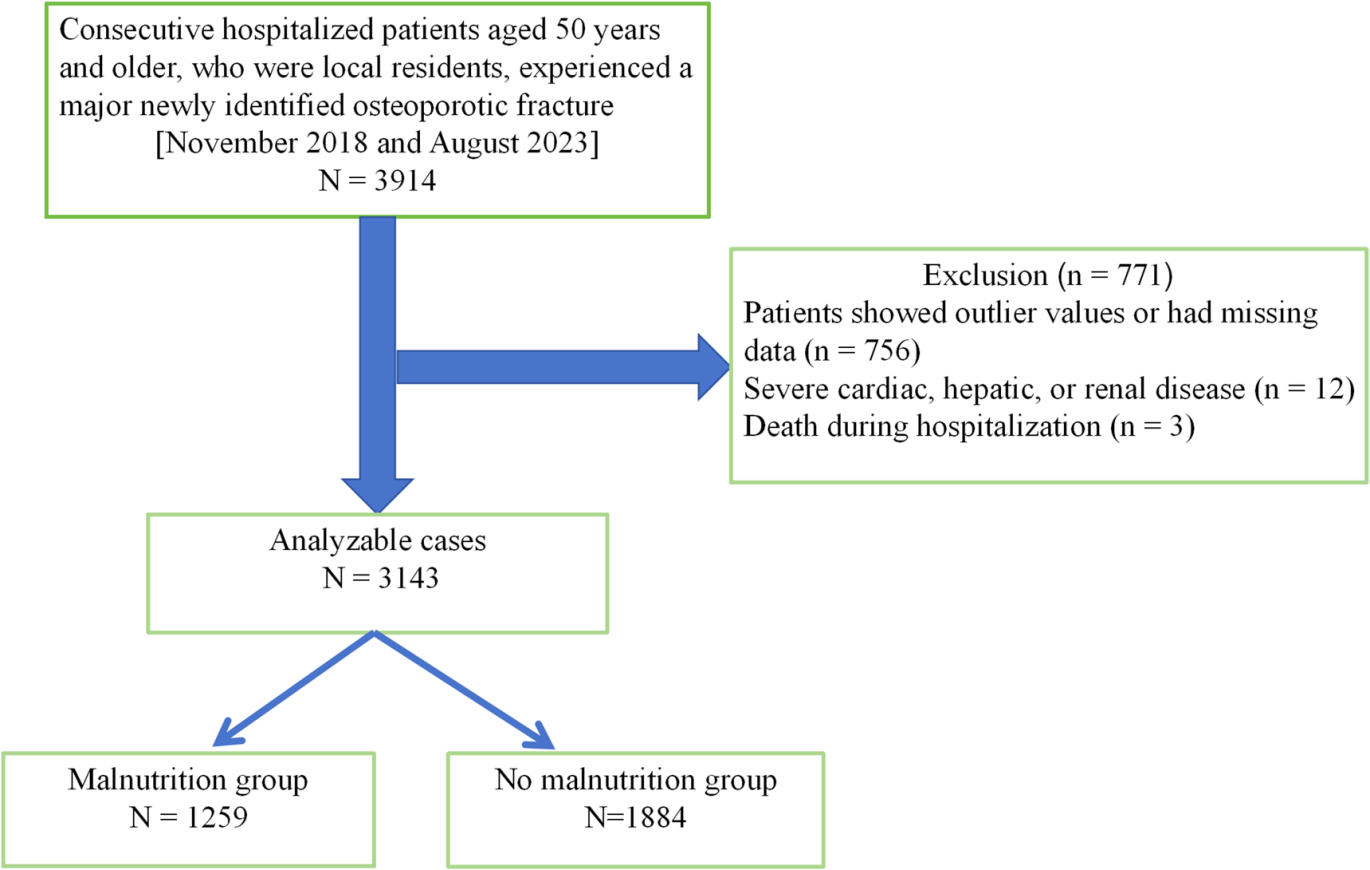
Study flow chart

Participation in this study was available throughout the entire duration of the research. Follow-up evaluations were carried out until December 31, 2023, as long as the follow-up period was longer than 24 h. The research enrolled 3914 sequentially admitted patients aged 50 and above who were newly diagnosed with significant OPFs. Figure 1 illustrates that this study encompassed participants aged 50 and older from 2018 to 2023, with a total of 3914 individuals. Among these participants, 771 were excluded. The inclusion criteria were: (1) individuals aged 50 years or older, and (2) follow-up time of 24 h or more. The exclusion criteria were: (1) Patients showed outlier values or had missing data (n = 756); (2) severe cardiac, hepatic, or renal disease (n = 12); (3) Death during hospitalization (n = 3) [12]. Outlier values might arise due to measurement inaccuracies, data entry errors, unusual situations, or true anomalies. Missing data included information on height, weight, and serum albumin levels.

### Study exposure variable and outcomes

The GNRI is a dietary evaluation index that has become widely recognized recently because of its ease of use and significant predictive value for different medical and surgical patient groups [13]. The GNRI is determined with the formula: 1.489 × serum albumin levels (g/L) + 41.7 × (body weight [kg]/ideal body weight) [14]. The ideal body weight is computed using the formula: 22 × height (meters) squared. This approach is associated with the lowest mortality rate and has been validated through research. If a patient’s weight surpasses the ideal weight, the weight-to-ideal weight ratio is adjusted to 1 [14, 15]. The GNRI > 98 indicates no malnutrition, whereas the GNRI ≤ 98 indicates malnutrition [16]. The main result measured in this study was mortality from any cause.

### Covariate variables

In this study, the recorded covariates included age, sex, hypertension, diabetes, hemoglobin, neutrophils, lymphocytes, monocytes, American Society of Anesthesiologists (ASA) category (1/2/3–4), and Charlson Comorbidity Index (CCI) score category (0/1/ ≥ 2). Baseline hemoglobin was assessed using the Sysmex XN-10 hematology analyzer (Sysmex Corporation, Kobe, Japan) with the SLS-Hb (Sodium Lauryl Sulfate-Hemoglobin) method. The Sysmex XN-10 hematology analyzer, utilizing flow cytometry with nuclear staining, was used to measure neutrophils, monocytes, and lymphocytes. The ASA category was assigned according to the anesthesiologist’s evaluation of the patient’s health status prior to the operation. Hospitalized patients were grouped into various categories based on the seriousness of their medical conditions and the potential effects on anesthesia management [17]. The CCI assigns values based on both the severity and quantity of comorbid conditions. A weighting of zero reflects no significant comorbidities that raise mortality risk, while higher weightings indicate a greater risk of mortality. The comorbidities taken into account included cardiovascular disease, diabetes, malignancy, kidney disease and other conditions. Each condition was allocated a score from 1 to 6, where higher scores denote a higher potential influence on mortality risk. The individual scores were aggregated to determine the patient’s overall CCI score [18, 19]. The ASA score was used to evaluate the patient’s overall health status, while the CCI score assessed comorbidities. All clinical metrics were reviewed within the first three days following hospital admission.

### Statistical analyses

Data on patient demographics, laboratory results, and clinical outcomes are reported either as medians with interquartile ranges (25th and 75th percentiles) or as averages with standard deviations (SD). The data are shown as counts and their respective percentages for each category. Non-normally distributed data were analyzed using Mann–Whitney U tests, whereas independent two-tailed t-tests were used for comparing data that followed a normal distribution. Chi-square tests were used to evaluate differences in categorical data, which were reported as counts and percentages. If the Chi-square test assumptions were not satisfied, the Fisher exact test was used as an alternative.

Each individual was classified into one of two categories: no malnutrition or malnutrition. In order to examine the relationship between various nutritional conditions and overall mortality, we utilized Kaplan–Meier curves to determine cumulative survival rates for overall mortality, and applied Cox proportional hazards regression models to explore the distinct correlation between different nutritional conditions and overall mortality, while adjusting for covariate influences. The outcomes of Model 1, Model 2, and Model 3 were analyzed for comparison. Initially, collinearity assessments were carried out utilizing variance inflation factor (VIF) evaluations. The need for adjusting covariates was subsequently assessed based on the following standards: Criterion 1 required either adding a confounding variable to the foundational model (which initially encompassed solely the GNRI (GNRI ≤ 98/GNRI > 98) and overall mortality without any additional variables) or excluding it from the comprehensive model (the comprehensive model comprised all probable confounding variables like age, sex, hypertension, diabetes, hemoglobin, neutrophils, lymphocytes, monocytes, ASA category, and CCI score category). This adjustment sought to achieve a minimum alteration of 10% in the adjusted odds ratio (OR). Criterion 2 involved either satisfying the conditions of the first criterion or identifying a covariate with a *P*-value less than 0.1 in the univariate analysis. In the initial model, no adjustments were made. However, the model 2 was modified to account for age, sex, and hypertension. In contrast to the previous Model 2, the subsequent Model 3 incorporated further modifications, which were determined by either satisfying the first criterion or the second criterion. In Model 3, modifications were made to account for factors including age, sex, hypertension, diabetes, hemoglobin, neutrophil, lymphocyte, monocyte, ASA category, and CCI score category. A smoothed line was utilized to assess both linear and non-linear correlations. The reliability of the research and differences among patient groups were assessed using subgroup analyses, in which patients admitted to the hospital were categorized based on particular variables. The researchers conducted additional analyses using the LRT to examine the interplay and changes within the subgroups.

The R packages^1^ and Empower Stats ^2^ were utilized for conducting all data analyses. A threshold of (*P* < 0.05) was applied for statistical significance, employing a two-sided test.

## Results

### Clinical characteristics

After screening, a total of 3,143 potentially eligible hospitalized patients with OPFs were enrolled (Fig. 1). Based on the data provided in Table 1, the patients who were admitted to the hospital were classified into two distinct categories: No Malnutrition Group (GNRI > 98): 1,884 cases, Malnutrition Group (GNRI ≤ 98): 1,259 cases. The mean age of the participants was 67.96 ± 11.04 years, with approximately 68% being female. Patients admitted to the hospital with nutritional deficiencies were older and had a higher likelihood of being female. Additional information on the initial characteristics of the study group is available in Table 1.

**Table 1:** Baseline characteristics of the patients

Characteristics	Mean ± SD	Mean ± SD	Mean ± SD	*P*-value
	Total	No malnutrition^a^	Malnutrition^b^	
	(n = 3143)	(n = 1259)	(n = 1884)	
Age, years	67.96 ± 11.04	66.46 ± 10.42	70.20 ± 11.55	< 0.001
Height, cm	160.56 ± 7.42	160.82 ± 7.22	160.18 ± 7.68	0.018
BMI, kg/m^2^	23.16 ± 3.35	23.87 ± 2.98	22.09 ± 3.59	< 0.001
Lymphocyte, 10^9/L	1.21 ± 0.53	1.25 ± 0.52	1.14 ± 0.55	< 0.001
Monocyte, 10^9/L	0.52 ± 0.31	0.49 ± 0.32	0.56 ± 0.29	< 0.001
Neutrophil, 10^9/L	6.78 ± 3.22	6.95 ± 3.17	6.53 ± 3.28	< 0.001
Albumin, g/L	39.68 ± 4.43	42.27 ± 2.76	35.82 ± 3.56	< 0.001
Hemoglobin, g/L	123.68 ± 18.33	130.03 ± 14.70	114.18 ± 19.12	< 0.001
Sex, N (%)				0.816
Female	2147(68.31%)	1284(68.15%)	863(68.55%)	
Male	996(31.69%)	600(31.85%)	396(31.45%)	
Hypertension, N (%)				0.210
No	2752 (87.56%)	1661 (88.16%)	1091 (86.66%)	
Yes	391 (12.44%)	223 (11.84%)	168 (13.34%)	
Diabetes, N (%)				0.097
No	3034 (96.53%)	1827 (96.97%)	1207 (95.87%)	
Yes	109 (3.47%)	57 (3.03%)	52 (4.13%)	
Fracture category, N (%)				< 0.001
Thoracic vertebra	531 (16.89%)	317 (16.83%)	214 (17.00%)	
Lumbar vertebra	933 (29.69%)	569 (30.20%)	364 (28.91%)	
Wrist	315 (10.02%)	217 (11.52%)	98 (7.78%)	
Proximal humerus	490 (15.59%)	329 (17.46%)	161 (12.79%)	
Femoral neck	874 (27.81%)	452 (23.99%)	422 (33.52%)	
ASA category, N (%)				< 0.001
1	442 (14.06%)	281 (14.92%)	161 (12.79%)	
2	2062 (65.61%)	1277 (67.78%)	785 (62.35%)	
3–4	639 (20.33%)	326 (17.30%)	313 (24.86%)	
CCI score category, N (%)				< 0.001
0	2843 (90.45%)	1733 (91.99%)	1110 (88.17%)	
1	232 (7.38%)	113 (6.00%)	119 (9.45%)	
≥ 2	68 (2.16%)	38 (2.02%)	30 (2.38%)	

*SD* standard deviation, *BMI* body mass index, *GNRI* Geriatric Nutritional Risk Index, *ASA* American Society of Anesthesiologists, *CCI* Charlson comorbidity index

^a^No malnutrition: GNRI > 98

^b^Malnutrition: GNRI ≤ 98

### Uncorrected and corrected Cox proportional risk regression models

Table 2 shows the relationship between the GNRI and overall mortality, with adjustments made at two stages using covariance analysis. Model 1 illustrates the analysis without any adjustments, whereas Model 2 incorporates adjustments for age, sex, and hypertension. Model 3 introduces additional adjustments for hemoglobin, diabetes, neutrophils, lymphocytes, monocytes, ASA category, and CCI score category. In the fully adjusted Model 3, the multivariate Cox regression analysis indicated that every 1-point rise in the GNRI corresponded to a 4% decrease in the risk of all-cause mortality (HR = 0.96, 95% CI 0.94, 0.98, *P*-value < 0.001). The study participants were divided into groups according to their GNRI values, separating individuals with malnutrition from those without. Individuals in the normal nutritional condition displayed a markedly lower long-term risk of overall mortality compared to those in the malnourished group. In both the unmodified Model 1 and the revised Model 2, malnutrition was notably connected to an elevated likelihood of death from any cause [HR for malnutrition: unadjusted Model 1: 1.93 (95% CI 1.50, 2.49, *P*-value < 0.001), Model 2: 1.34 (95% CI 1.04, 1.74, *P*-value = 0.026)]. Furthermore, in the fully adjusted Model 3, the aHR for malnutrition was 1.42 (95% CI 1.05, 1.90, *P-*value = 0.021).

**Table 2:** Cox proportional hazard ratios (95% Confidence Intervals) for all-cause mortality

Status	Model 1^a^		Model 2^b^		Model 3^c^	
	HR (95%CI)	*P*-value	HR (95%CI)	*P*-value	HR (95%CI)	*P*-value
Continuous						
GNRI (per 1 score)	0.95 (0.94, 0.97)	< 0.001	0.97 (0.96, 0.99)	0.001	0.96 (0.94, 0.98)	< 0.001
Categories						
No malnutrition	Reference		Reference		Reference	
Malnutrition	1.93 (1.50, 2.49)	< 0.001	1.34 (1.04, 1.74)	0.026	1.42 (1.05, 1.90)	0.021

*GNRI* Geriatric Nutritional Risk Index, *ASA* American Society of Anesthesiologists, *CCI* Charlson Comorbidity Index

^a^No adjustment

^b^Adjusted for age, sex, hypertension

^c^Adjusted for age, sex, hypertension, diabetes, hemoglobin, lymphocyte, monocyte, neutrophil, ASA category, CCI score category

### Smoothed curve fitting and threshold analyses

**Fig. 2:**
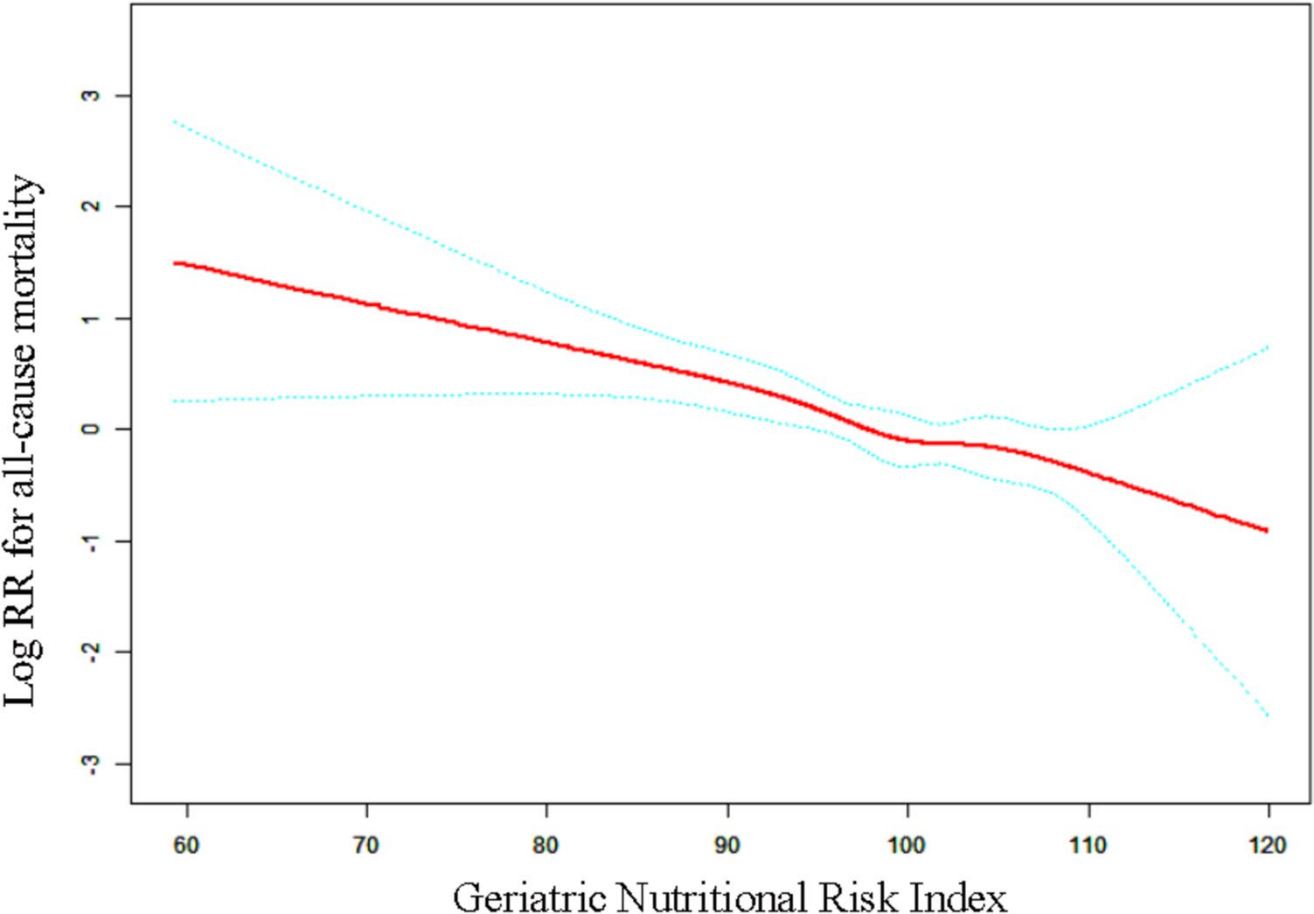
Adjusted smoothed curves corresponding to the relationship between the GNRI and overall mortality among inpatients with OPFs. The red curve in the middle represents the estimated value, and the blue curves on either side represent the 95% CI. The adjusted factors were age, sex, hypertension, hemoglobin, diabetes lymphocyte, monocyte, neutrophil, ASA category, and CCI score category. *GNRI* Geriatric Nutritional Risk Index, *ASA* American Society of Anesthesiologists, *CCI* Charlson Comorbidity Index, *CI* confidence interval

In hospitalized patients with OPFs, a linear relationship between the GNRI and mortality from any cause was observed after accounting for confounding factors including age, sex, hypertension, diabetes, hemoglobin, neutrophil, lymphocyte, monocyte, ASA category, and CCI score category (Fig. 2). The threshold effect examination in Model 3, which explores the link between the GNRI and overall mortality, is summarized in Table 3. The examination demonstrated a linear correlation between the GNRI levels and overall mortality among inpatients with OPFs, as evidenced by the *P*-value obtained from LRT (logarithmic likelihood ratio test) = 0.367. Significantly, we observed a significantly stronger negative relationship with overall mortality (HR = 0.96; 95%CI 0.94, 0.98; *P*-value < 0.001). The data suggests that for every 1-point rise in the GNRI, there is a corresponding 4% decrease in the likelihood of mortality from any cause.

**Table 3:** Threshold analyses exploring the association between the GNRI and all-cause mortality

	Model 3^a^
	HR (95% CI) *P*-value
Model A^b^	
One line effect	0.96 (0.94, 0.98) < 0.001
Model B^c^	
GNRI turning point (K), per 1 score	86.71
< K	0.99 (0.93, 1.05) 0.689
> K	0.96 (0.93, 0.98) < 0.001
Slope 2–slope 1	0.97 (0.90, 1.04) 0.384
LRT^d^	0.367

*GNRI* Geriatric Nutritional Risk Index, *HR* hazard ratio, *CI* confidence interval, *ASA* American Society of Anesthesiologists, *CCI* Charlson Comorbidity Index, *LRT* logarithmic likelihood ratio test

^a^Adjusted for age, sex, hypertension, hemoglobin, diabetes, neutrophil, lymphocyte, monocyte, ASA category, and CCI score category

^b^Linear analysis, *P*-value < 0.05 indicates a linear relationship

^c^Nonlinear analysis

^d^*P*-value > 0.05 suggests that Model A substantially varies from Model B, implying a direct correlation

### Analysis of the Kaplan–Meier survival curves based on GNRI levels

**Fig. 3:**
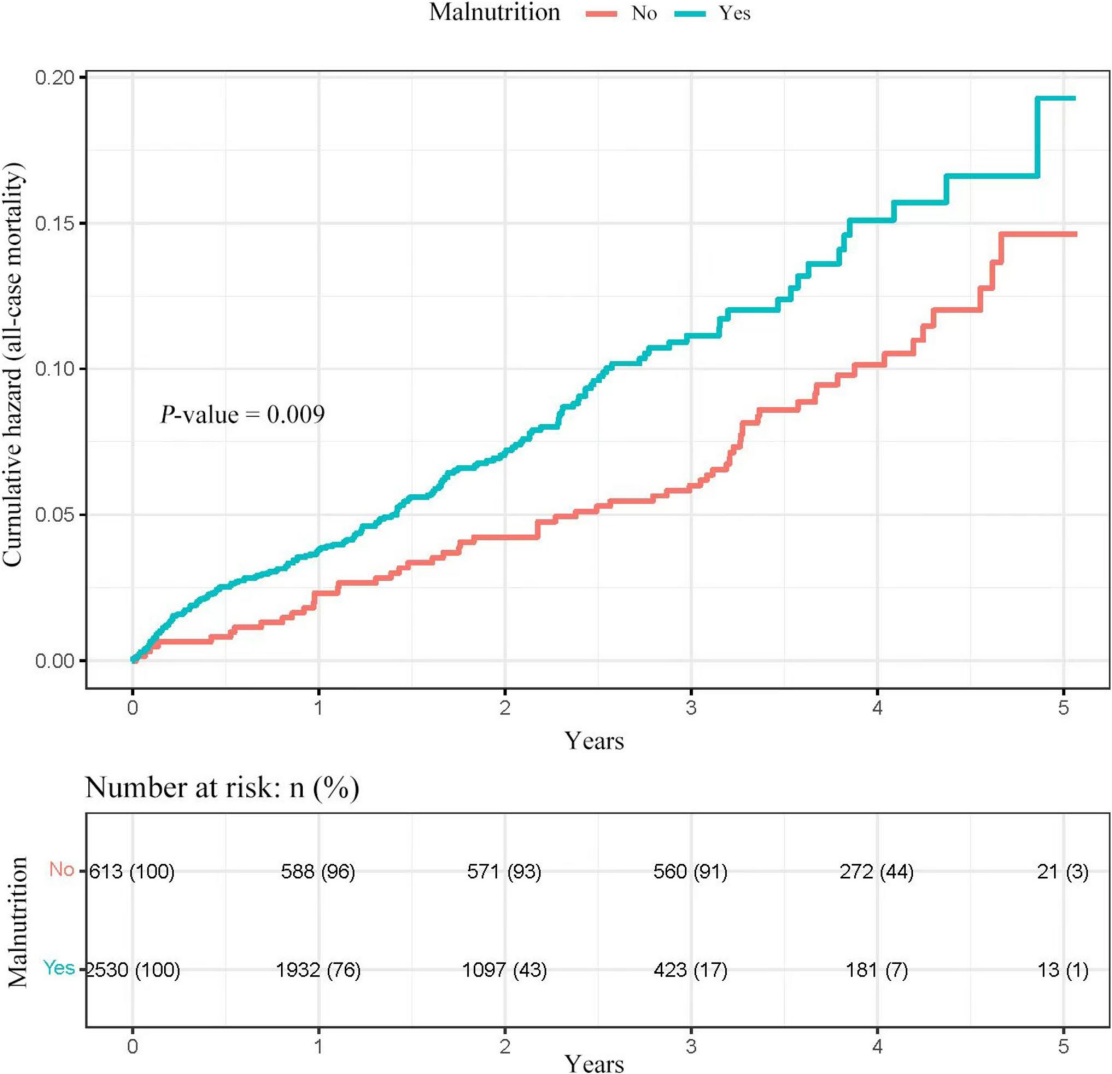
Kaplan–Meier curves for estimating the cumulative risk of mortality of patients in the malnutrition (blue line) and no malnutrition (red line) group. The adjusted factors were age, sex, hypertension, hemoglobin, diabetes lymphocyte, monocyte, neutrophil, ASA category, and CCI score category. *GNRI* Geriatric Nutritional Risk Index, *ASA* American Society of Anesthesiologists, *CCI* Charlson Comorbidity Index, *CI* confidence interval

The patient cohort was categorized into two categories according to the GNRI: one group with malnutrition (GNRI ≤ 98) and another group without malnutrition (GNRI > 98). We assessed the association between the GNRI levels and the cumulative risk of mortality by analyzing Kaplan–Meier curves (Fig. 3). The findings indicated an inverse relationship between the GNRI levels and the cumulative risk of mortality, with a notably greater risk observed in the malnutrition group relative to the no malnutrition group (*P*-value = 0.009).

### Stratified analysis in subgroups

**Fig. 4:**
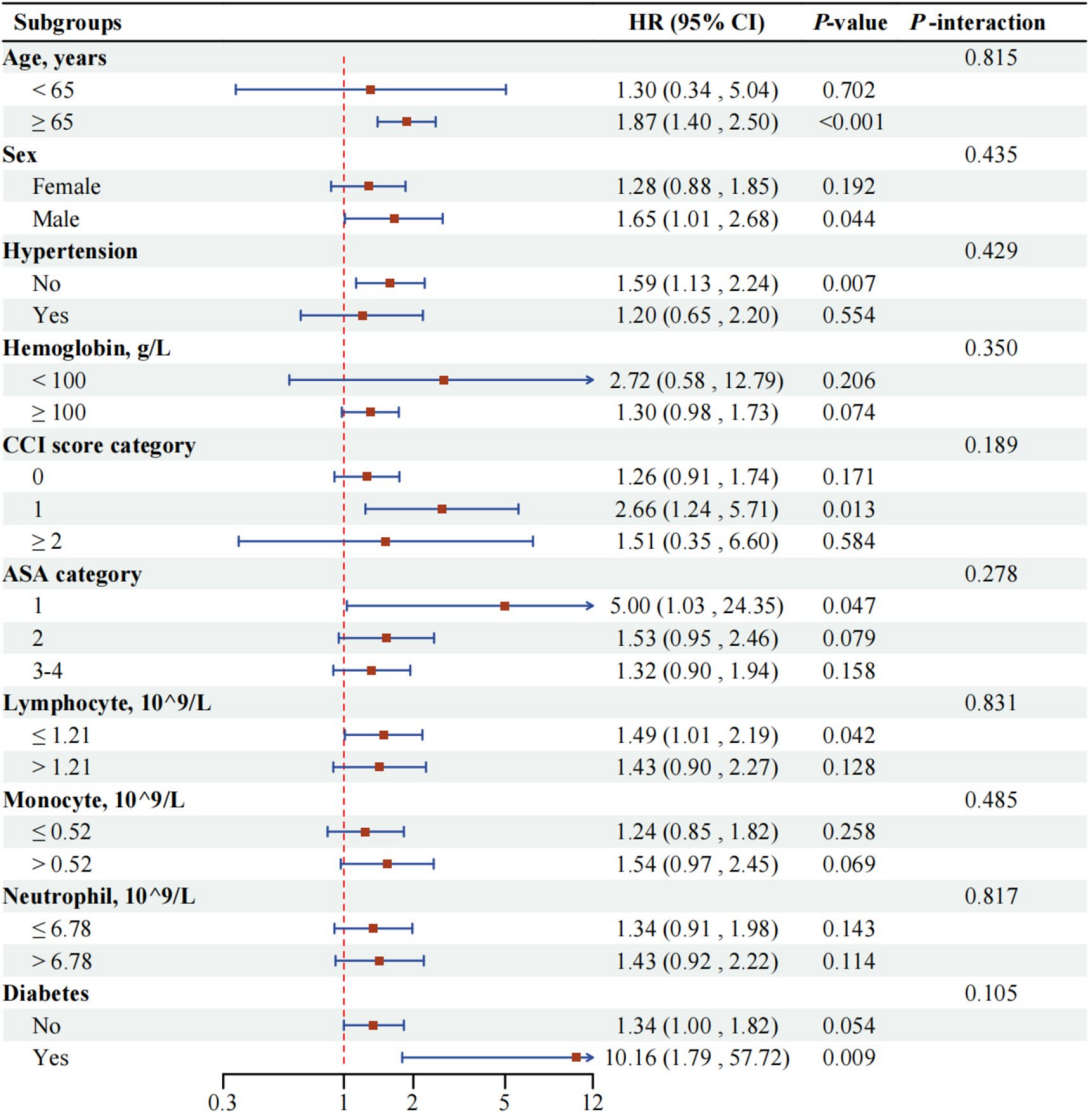
Forest plots for stratification analyses in subgroups. The stratified analysis assesses the strength of the noted correlation between the GNRI levels and overall mortality rates. *GNRI* Geriatric Nutritional Risk Index, *CI* confidence interval, *HR* hazard ratio

Subgroup analyses were subsequently conducted based on patient age, sex, CCI score category, hemoglobin, hypertension, diabetes, neutrophils, lymphocytes, monocytes, and ASA category to assess the strength of the noted correlation between the GNRI levels and overall mortality rates (Fig. 4). These analyses were performed using a fully adjusted multivariate Cox regression model, controlling for all variables except the subgroup variable under consideration. The results demonstrated no significant interactions across the stratified subgroups (*P-*interaction > 0.05), confirming the consistency of the association across different patient characteristics.

## Discussion

This research signifies an initial inquiry conducted at the population level, focusing on exploring the link between the GNRI and overall mortality in hospitalized individuals with diagnosed OPFs. In this study, nearly two-fifths of the osteoporotic population were classified as malnourished based on the GNRI scores. At the outset of our research, a clear linear correlation was established between the GNRI and mortality from all causes. Specifically, our findings demonstrated that for every increment of 1 point in GNRI, there was a corresponding 4% decrease in the likelihood of all-cause mortality. Furthermore, when participants were categorized according to their GNRI levels, the group identified as malnourished exhibited a 42% increased risk of all-cause mortality in comparison to the group without malnutrition. These results indicate that the GNRI may could serve as an indicator of mortality among hospitalized patients with OPFs, and that malnutrition might be a changeable risk factor that adds to the heightened mortality risk in this group.

Nutritional status is a crucial determinant of mortality and adverse outcomes across a range of clinical conditions [20, 21], particularly in chronic diseases such as hypertension, diabetes, cardiovascular disease, and osteoporosis [22–24]. The GNRI functions as a crucial marker for evaluating nutritional status in older populations. This index is derived from measurements of serum albumin levels, body weight, and height. Furthermore, the GNRI includes a combined assessment of serum albumin levels and BMI, which enhances its diagnostic accuracy and increases its effectiveness in clinical settings [25].

The GNRI has been extensively employed in many clinical investigations to evaluate the nutritional condition of hospitalized patients and has been confirmed as an important prognostic indicator across different illnesses. Numerous research efforts have shown the effectiveness of the GNRI in assessing nutritional status and forecasting outcomes for hospitalized patients receiving hemodialysis in Asia [26, 27]. Xia Shen and colleagues conducted a cohort study utilizing NHANES data, which highlighted the GNRI as a standalone prognostic marker for patients with diabetes who are hospitalized [28]. Furthermore, a retrospective group analysis proposed that the GNRI might act as a solitary prognostic factor for patients hospitalized with severe diabetic foot ulcers [29]. Recently, a study from Japan by T. Tsutsui and colleagues utilized data from a multicenter prospective registry in the northern Kyushu district to evaluate the GNRI as a predictive marker in older hospitalized patients with fragility hip fractures [12]. Our study, which analyzed hospitalized patients with primary OPFs from Kunshan Hospital, affiliated to Jiangsu University, reached similar conclusions. It was observed that the GNRI may function as a predictive indicator for elderly patients admitted to the hospital with OPFs (HR = 0.96 *P*-value < 0.001). Our research underscores a significant link between the GNRI and overall mortality in patients with OPFs who are hospitalized. Patients in the hospital who are malnourished (GNRI ≤ 98) face a mortality risk 1.44 times higher than those who are not malnourished (GNRI > 98) (95% CI 1.07, 1.93).

Serum albumin serves as a vital indicator of nutritional status [30], with decreased levels being closely associated with aging and increased mortality among the elderly population [31]. Consequently, low serum albumin levels are regarded as an indicator of increased risk for death. An increasing volume of evidence indicates that reduced serum albumin levels are linked to a heightened risk of osteoporosis [32]. Reduced levels of albumin have been linked to a higher likelihood of brittle bone fractures among patients in the hospital receiving peritoneal dialysis [33]. Study indicates that a blood albumin concentration below 3.8 g/dL (or a decrease in serum albumin levels) is associated with an increased risk of death in individuals suffering from renal failure [34]. BMI is now acknowledged as a measure for assessing nutrition. It is well established that a low BMI is associated with an increased risk of various types of fractures [35]. Additionally, serum albumin and BMI are key components of the GNRI, which may elucidate the observed link between the GNRI scores and death in individuals with OPFs.

Nutritional status plays a significant role in bone health. Adequate nutrition is vital for maintaining bone density. Studies have demonstrated that higher GNRI scores are positively correlated with BMD-T scores, indicating that better nutritional status contributes to improved bone quality [36]. The maintenance of bone density through adequate nutrition can significantly reduce the risk of fractures, thereby decreasing mortality associated with fall-related injuries. Elderly patients often face chronic metabolic diseases such as diabetes and hypertension, which can negatively impact nutritional status and, consequently, bone health. Malnutrition resulting from these conditions can lead to decreased bone density and increased fracture risk [37]. The presence of chronic conditions can significantly impact nutritional status and markedly increase mortality risk, creating a vicious cycle that exacerbates health issues [38]. Kumagai et al. found that a higher GNRI score is associated with lower rates of hospitalization and complications, which is directly linked to reduced mortality rates [39].

Inadequate nutrition, especially lack of proteins and vital micronutrients, weakens the immune system, leading to a marked rise in vulnerability to infections. For instance, individuals suffering from severe protein-energy malnutrition (PEM) exhibit a higher incidence of severe infections, such as pneumonia and sepsis, which are prominent causes of death in this population [40]. Mechanistically, nutritional deficiencies impair the production and effectiveness of immune cells, such as T-cells and B-cells [41]. Essential nutrients like proteins, zinc, and vitamin A are critical for the synthesis of immunoglobulins and cytokines, which are vital for a robust immune response. In the absence of sufficient nutrition, the synthesis of these immune elements is reduced, resulting in a weakened capability to resist infections and a heightened risk of death [41, 42].

Inadequate nutrition causes significant metabolic disturbances and contributes to multi-organ dysfunction, further elevating mortality risk. The most prevalent nutritional shortfall is a lack of vitamin D [43]. Extended shortages of vitamin D can result in significant bone weakening and a higher likelihood of fractures, which may lead to severe and potentially fatal complications [44]. Lower levels of vitamin D have been associated with increased blood pressure. Earlier research has suggested that a deficiency in vitamin D is linked to a greater occurrence of hypertension and is inversely correlated with diastolic blood pressure [45]. Hypertension is one of the leading contributors to mortality globally [46]. Additionally, research has shown that a lack of vitamin D is common among individuals with cardiac blood vessel disorder or heart dysfunction. Numerous observational studies have found that reduced levels of 25(OH)D are linked to a higher risk of overall mortality and readmission due to cardiac failure [47, 48].

The identification of the GNRI as a possible indicator of overall mortality in individuals with OPFs carries significant clinical consequences. The GNRI, which indicates both nutritional condition and the seriousness of existing health issues, provides a practical means for doctors to evaluate the risk of negative outcomes in this at-risk group. Since malnutrition is a risk factor that can be addressed and improved, early identification of at-risk individuals through the GNRI could lead to timely nutritional interventions that may improve overall survival rates. Additionally, incorporating the GNRI into standard clinical practice may improve patient stratification, facilitating more tailored treatment strategies. Patients who are flagged with a low GNRI score might require enhanced nutritional support, more vigilant monitoring, and potentially more intensive management of osteoporosis and its related issues. Taking this proactive stance could help lower the risk of complications like infections, slow fracture recovery, and additional fractures, all of which can lead to higher mortality rates. Beyond its use in managing individual patients, the GNRI could act as a useful measure in clinical research and epidemiological investigations, offering a consistent way to assess how nutritional interventions affect survival rates. Future research should focus on refining GNRI cut-off values specific to OPF patients and validating its predictive accuracy across diverse patient populations and healthcare settings. To sum up, integrating the GNRI into the clinical evaluation of patients with OPFs could not only boost patient outcomes through tailored interventions but also improve the overall management of this vulnerable group by pinpointing those who are most likely to gain from improved nutritional and clinical care approaches.

This research features several notable advantages, such as a substantial sample size and a prolonged follow-up duration, both of which contribute significantly to the reliability of our results. The study cohort, which accurately reflects the older demographic of Chinese individuals with OPFs, enhances the external validity of the findings. Additionally, the open enrollment methodology reduces selection bias and guarantees a varied participant group. The prolonged follow-up allowed for a thorough analysis of mortality rates across different nutritional statuses, providing valuable insights into long-term outcomes in this high-risk group. However, there are limitations to consider. The research concentrated exclusively on overall mortality as the primary outcome, which, although objective and clinically relevant, could restrict the breadth of the conclusions drawn. Furthermore, we identified only GNRI as an indicator of malnutrition and did not investigate other indicators. However, using body weight and serum albumin, GNRI has been evaluated as a simple and accurate method for assessing malnutrition. This retrospective cohort study primarily identifies associations within historical data and inherently cannot establish causality. Due to its observational nature, our findings are limited to correlations, with potential biases such as selection and information bias affecting their validity. Although we have adjusted for known confounders, the influence of unmeasured variables cannot be excluded. These factors should be considered when interpreting the results, and future prospective studies are needed to clarify causal relationships. Finally, the exclusive focus on one center in the study could restrict the applicability of findings to diverse ethnic populations. To enhance the generalizability and robustness of these results, future studies should incorporate comprehensive, multicenter randomized controlled trials involving varied populations.

## Conclusions

This research indicates that reduced GNRI levels are closely linked to a higher risk of overall mortality in patients hospitalized with OPFs. These results highlight the critical need for nutritional risk assessment and the adoption of preventive measures within this patient group. To lower the risk of early mortality, these patients are encouraged to sustain a well-balanced diet. Clinicians should be proactive in evaluating patients’ nutritional status and offering timely and suitable dietary advice. The dynamic monitoring of the GNRI in clinical applications is essential for optimizing patient outcomes. Nonetheless, additional studies are needed to clarify the precise thresholds and the applicability of the GNRI in various patient groups.

## Supplementary Information

Below is the link to the electronic supplementary material.

Supplementary file1 (XLSX 407 KB)

Supplementary file2 (DOCX 18 KB)

## Data Availability

Data is provided within the supplementary information files.
