# Supplementary material for: Association between Geriatric Nutritional Risk Index and all-cause mortality in individuals with osteoporotic fractures: a retrospective cohort study
Source: Aging Clin Exp Res. 2025 Mar 11;37(1):77. doi: 10.1007/s40520-025-02978-w (PMC11897076; doi:10.1007/s40520-025-02978-w)
Supplement: Supplementary file 2 — Supplementary file2 (DOCX 18 KB) [file 40520_2025_2978_MOESM2_ESM.docx]

**Table 1s**. Baseline characteristics of the patients

| **Characteristics** | **Mean ± SD** | **Mean ± SD** | **Mean ± SD** | ***P*-value** |
| --- | --- | --- | --- | --- |
|  | **Total** | **No malnutrition^a^** | **Malnutrition^b^** |  |
| Calcium, mmol/L | 2.19 ± 0.13 | 2.23 ± 0.11 | 2.13 ± 0.14 | <0.001 |
| Vitamin D, ng/ml | 18.44 ± 7.50 | 18.83 ± 6.83 | 17.95 ± 8.28 | 0.018 |
| Lumber BMD, g/cm² | 0.74 ± 0.15 | 0.75 ± 0.15 | 0.73 ± 0.15 | <0.001 |
| Troch BMD, g/cm² | 0.53 ± 0.11 | 0.54 ± 0.11 | 0.51 ± 0.11 | <0.001 |
| Inter BMD, g/cm² | 0.83 ± 0.18 | 0.85 ± 0.17 | 0.80 ± 0.18 | <0.001 |
| Neck BMD, g/cm² | 0.61 ± 0.13 | 0.62 ± 0.12 | 0.59 ± 0.13 | <0.001 |

^a^No malnutrition: GNRI > 98

^b^Malnutrition: GNRI ≤ 98

Abbreviations: SD standard deviation, BMD, Bone Densitometry.
